# Cisplatin resistance by induction of aldo-keto reductase family 1 member C2 in human bladder cancer cells

**DOI:** 10.3892/ol.2013.1768

**Published:** 2013-12-19

**Authors:** AKITOMI SHIRATO, TADAHIKO KIKUGAWA, NORIYOSHI MIURA, NOZOMU TANJI, NOBUAKI TAKEMORI, SHIGEKI HIGASHIYAMA, MASAYOSHI YOKOYAMA

**Affiliations:** 1 Department of Urology, Ehime University Graduate School of Medicine, Toon, Ehime 791-0295, Japan; 2 Proteomics Core-Laboratory, Proteo-Medicine Research Center, Ehime University Graduate School of Medicine, Toon, Ehime 791-0295, Japan; 3 Department of Biochemistry and Molecular Genetics, Ehime University Graduate School of Medicine, Toon, Ehime 791-0295, Japan; 4 Department of Cell Growth and Tumor Regulation, Proteo-Medicine Research Center, Ehime University Graduate School of Medicine, Toon, Ehime 791-0295, Japan

**Keywords:** bladder cancer, cisplatin resistance, aldo-keto reductase family 1 member C2

## Abstract

Cisplatin is currently the most effective anti-tumor agent available against bladder cancer. To clarify the mechanism underlying cisplatin resistance in bladder cancer, the present study examined the role of the aldo-keto reductase family 1 member C2 (AKR1C2) protein on chemoresistance using a human bladder cancer cell line. The function of AKR1C2 in chemoresistance was studied using the human HT1376 bladder cancer cell line and the cisplatin-resistant HT1376-CisR subline. AKR1C2 was expressed in HT1376-CisR cells, but not in the parental cells. The effect of small interfering (si) RNAs and an inhibitor targeting AKR1C2 was examined to determine whether cisplatin sensitivity can be rescued by blocking AKR1C2 expression or function. Silencing of AKR1C2 mRNA or inhibition of AKR1C2 by 5β-cholanic acid resulted in a decrease in the survival of cells following cisplatin exposure. Intracellular accumulation of reactive oxygen species (ROS) was determined using a 2,7-dichlorodihydrofluorescein diacetate (H_2_DCFDA) fluorescent probe. Cisplatin exposure increased the level of intracellular ROS in HT1376 cells in a dose-dependent manner. The ROS levels in HT1376-CisR cells were significantly lower than those in HT1376 cells and knockdown of AKR1C2 mRNA significantly restored ROS levels. Cisplatin exposure did not increase intracellular ROS in HT1376-CisR cells, although the level of intracellular ROS increased in HT1376 cells following cisplatin exposure. Silencing of AKR1C2 mRNA restored the ROS increase response to cisplatin and menadione as an oxidative stressor in HT1376-CisR cells. Menadione has the function of an oxidative stressor. The silencing of AKR1C2 mRNA restored the increased ROS response to cisplatin and menadione in HT1376-CisR cells. These results indicate that induction of AKR1C2 in human bladder cancer cells aids in the development of cisplatin resistance through antioxidative effects. The results of this study indicate that AKR1C2 may be an effective molecular target for restoring cisplatin resistance.

## Introduction

Urothelial carcinoma (UC) is a highly chemosensitive disease. Cisplatin is a key drug for the treatment of advanced or metastatic UC. To date, the combination of methotrexate, vinblastine, doxorubicin and cisplatin (M-VAC) has been accepted as the most effective therapy for metastatic UC (1). A randomized trial that was designed to compare a two-drug regimen comprising gemcitabine and cisplatin (GC) with M-VAC, revealed that GC provided a similar survival advantage to M-VAC but with improved safety and tolerability (2). However, the prognosis for patients with metastatic UC of the urinary tract remains poor even with GC treatment. From our experience with GC, the median time to progression and the median overall survival time for cisplatin-naïve patients were 6 and 14 months, respectively (3). In this study, the overall response rate to treatment for patients on this regimen was 63%, while 37% of the patients were completely or almost resistant to cisplatin. In addition, only 31% of the patients who relapsed >6 months after treatment with the prior cisplatin-based regimen exhibited an objective response to cisplatin. These results suggest that cancer cells naturally have, or eventually develop, cisplatin resistance. Therefore, the acquisition of chemoresistance remains a major obstacle in cancer treatment, which ultimately leads to mortality.

We previously established a cisplatin-resistant subline from the human HT1376 bladder cancer cell line (HT1376-CisR) to elucidate the possible mechanisms underlying cisplatin resistance in bladder cancer cells (4). Comparative proteomic analysis of HT1376 and HT1376-CisR cells has revealed 36 differentially-expressed proteins, of which 21 proteins are upregulated in HT1376-CisR cells (4). Among the differentially regulated proteins, aldo-keto reductase family 1 member C2 (AKR1C2) was markedly expressed in HT1376-CisR cells but not in HT1376 cells.

The AKR superfamily consists of nicotinamide adenine dinucleotide phosphate-dependent oxidoreductases that metabolize a wide range of endogenous and exogenous compounds. AKR overexpression has been associated with chemotherapy resistance in a variety of cancer cell lines (5–10). AKR overexpression is also associated with disease progression in bladder (11) and prostate cancer (12). Chen *et al* found that AKR overexpression, which induced resistance to chemotherapy, also reduced reactive oxygen species (ROS) production using human ovarian cancer cells (6). In contrast, no correlation between AKR expression and ROS levels was observed in lung cancer cells (8). Thus, the importance of AKRs in the mechanism of drug resistance remains unclear. In the present study, attempts were made to clarify the underlying cisplatin resistance mechanisms by analyzing the function of AKR1C2 at the cellular and molecular levels.

## Materials and methods

### Reagents

RPMI-1640 and fetal bovine serum (FBS) for cell culture were supplied by Life Technologies (Carlsbad, CA, USA). Cisplatin, 5β-cholanic acid and menadione were purchased from Sigma-Aldrich (Tokyo, Japan). 5β-cholanic acid and menadione were used as an AKR1C2 inhibitor and an oxidative stressor, respectively. 2,7-Dichlorodihydrofluorescein diacetate (H_2_DCFDA) was purchased from Life Technologies. Anti-AKR1C2 and anti-β-tubulin (loading control) rabbit polyclonal antibodies were obtained from NOVUS Biological (Littleton, CO, USA) and Abcam (Cambridge, UK), respectively.

### Cell culture

The human HT1376 bladder cancer cell line used in this study was purchased from DS Pharma Biomedical (Osaka, Japan). Cells were maintained in RPMI-1640 medium supplemented with 10% FBS in a humidified incubator at 37°C and 5% CO_2_. Cisplatin-resistant cells (HT1376-CisR) were obtained from the parental HT1376 cells using an intermittent stepwise selection protocol over 12 months, ending with exposure to 5 μM cisplatin (4).

### Western blot analysis

Cells were lysed with an ice-cold lysis buffer and protease inhibitor cocktail mix (Sigma-Aldrich). Samples were centrifuged at 12,000 × g for 10 min at 4°C and supernatants were electrophoresed by SDS-PAGE and transferred to polyvinylidene difluoride membranes (Millipore, Bedford, MA, USA). Following blocking with 5% skimmed milk, the membranes were probed with primary antibodies overnight at 4°C, followed by horseradish peroxidase-conjugated secondary antibody (GE Healthcare, Chalfont St. Giles, UK) for 1 h at room temperature. The immune complexes were visualized with the Enhanced Chemiluminescence Plus detection system (GE Healthcare) according to the manufacturer’s instructions.

### Drug cytotoxicity analysis

To analyze drug cytotoxicity, 1.0×10^4^ cells/well were cultured with concentrations of cisplatin graded between 0.5×10^−7^ and 10^−3^ M cisplatin in at least 3 replicate wells at 37°C. Following 72 h of cisplatin treatment, the cells were counted using a Scepter 2.0 Handheld Automated Cell Counter (Merck Millipore, MA, USA). Cell survival in the absence of cisplatin was defined as 100% cell survival. The drug concentration that resulted in 50% growth inhibition (IC_50_) was determined from the corresponding dose-response curve.

### Intracellular ROS accumulation

Intracellular ROS accumulation was determined using the method described by Tardito *et al* (13). H_2_DCFDA does not fluoresce but becomes fluorescent when it is hydrolyzed to H_2_DCF inside cells by nonspecific esterases. Briefly, the samples were plated in 96-well plates at a density of 4.0×10^4^ cells/well. Following overnight incubation with or without reagent, intracellular ROS was examined. Growth medium was removed and 100 μl prewarmed Hank’s balanced salt solution (HBSS; Life Technologies) containing 20 μM H_2_DCFDA was added at 37°C without exposure to light. The prewarmed HBSS with H_2_DCFDA was prepared fresh for each assay. Following incubation at 37°C for 30 min, the cells were washed twice with HBSS and ROS generation was measured as fluorescence intensity using a fluorescence multiplate reader (Flex Station 3; Molecular Devices, Sunnyvale, CA, USA) with an excitation wavelength of 480 nm and an emission wavelength of 530 nm.

### Technologies

The small interfering (si) RNA sequences were as follows: Sense, 5′-CGGCCGGAAAAGAAAGACATT-3′ and antisense, 5′-UGUCUUUCUUUUCCGGCCGAT-3′. For the control, the following non-targeting siRNA cocktails were used: 5′-ATCCGCGCGATAGTACGTA-3′, 5′-TTACGCTA GCGTAATACG-3′ and 5′-TATTCGCGCCTATAGCGGT-3′. The cells were transiently transfected with AKR1C2 siRNA and control siRNA using Lipofectamine RNAiMAX (Life Technologies) and Optimen I (Life Technologies) at a 120 pM concentration. Following 48-h incubation, cells were utilized for each assay.

### Statistical analysis

All values are expressed as the mean ± standard deviation of at least 3 independent experiments. The unpaired Student’s t-test was used for statistical analysis in this study. P<0.05 was considered to indicate a statistically significant difference.

## Results

**Figure 1:**
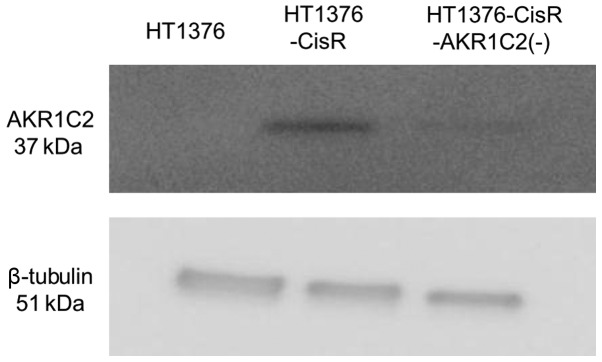
AKR1C2 protein expression in HT1376-CisR cells was markedly increased in comparison with the parental cells. AKR1C2 small interfering RNA reduced expression by ~80% in HT1376-CisR cells. AKR1C2 and β-tubulin exhibit discrete bands of the same molecular weight (AKR1C2, 37 kDa; β-tubulin, 51 kDa). AKR1C2, aldo-keto reductase family 1 member C2; CisR, cisplatin-resistant.

Expression levels of AKR1C2 protein levels were examined by western blot analysis (Fig. 1). Expression was detected in the HT1376-CisR cells, but in the parental cells. AKR1C2-siRNA reduced the AKR1C2 protein levels by ~80% in HT1376-CisR cells.

**Figure 2:**
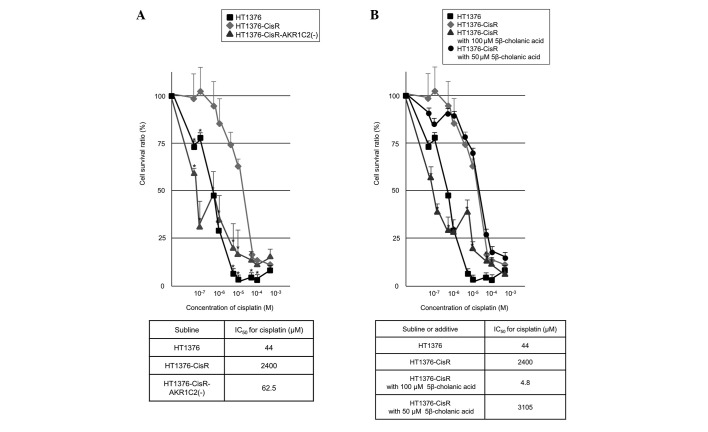
Effect of AKR1C2 expression on cisplatin IC_50_ values in parental and HT1376-CisR cells. Cells were treated with various cisplatin concentrations for 72 h, and then quantified using a cell counter. Each assay was performed in triplicate. Cell survival in the absence of cisplatin was set as 100%. (A) Silencing AKR1C2 restored HT1376-CisR cell response to cisplatin. (B) Inhibition of AKR1C2 by 100 μM 5β-cholanic acid restored the HT1376-CisR response to cisplatin. ^*^P<0.05, vs. HT1376-CisR. Bars indicate standard deviation. AKR1C2, aldo-keto reductase family 1 member C2; CisR, cisplatin-resistant.

Next, the effect of AKR1C2 expression on cell survival was examined. Fig. 2A shows the relative number of surviving HT1376 and HT1376-CisR cells following treatment with various concentrations of cisplatin. The IC_50_ values for cisplatin treatment in HT1376 and HT1376-CisR cells were 44 and 2,400 μM, respectively. The IC_50_ for HT1376-CisR was thus 54.5-fold higher than that of HT1376 cells, indicating that a cisplatin-resistant cell line was successfully established. AKR1C2-siRNA markedly rescued the cisplatin sensitivity of HT1376-CisR. The IC_50_ value for cisplatin treatment in HT1376-CisR cells transiently transfected with AKR1C2 siRNA [HT1376-CisR-AKR1C2(−)] was 62.5 μM.

Next, the inhibitory effect of 5β-cholanic acid on cell survival was examined. Fig. 2B shows the relative number of surviving HT1376-CisR cells following treatment with or without 5β-cholanic acid and various concentrations of cisplatin. All HT1376-CisR cells died following incubation for 72 h in medium with 150 μM 5β-cholanic acid, possibly due to its strong cytotoxicity. Addition of 100 μM 5β-cholanic acid to the medium restored the cisplatin response of HT1376-CisR cells, whereas 50 μM 5β-cholanic acid did not. The IC_50_ values for cisplatin treatment in HT1376-CisR cells cultured at concentrations of 50 and 100 μM 5β-cholanic acid were 3,105 and 4.8 μM, respectively. These results indicate that AKR1C2 plays an important role in cisplatin resistance in HT1376 cells.

**Figure 3:**
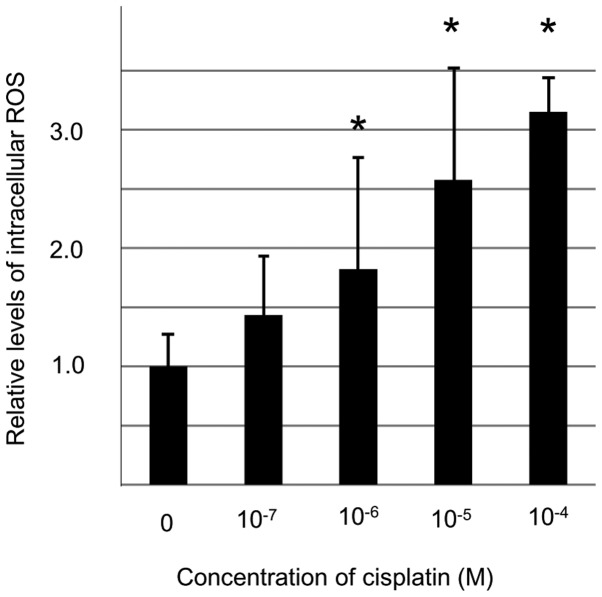
Effect of cisplatin on intracellular ROS in HT1376 cells. Exposure to cisplatin increased the levels of intracellular ROS in HT1376 cells in a dose-dependent manner. ^*^P<0.05, vs. HT1376 cells cultured without cisplatin. Bars indicate standard deviation. ROS, reactive oxygen species.

**Figure 4:**
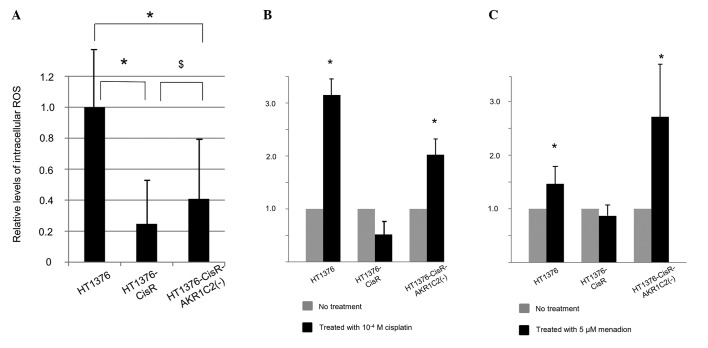
Relative values of intracellular ROS measured using a 2,7-dichlorodihydrofluorescein diacetate probe. (A) Basal intracellular ROS levels in HT1376, HT1376-CisR and HT1376-CisR cells transiently transfected with AKR1C2 small interfering RNA [HT1376-CisR-AKR1C2(−)]. ^*^P<0.05 and ^$^P<0.05, vs. HT1376 and HT1376-CisR cells cultured without cisplatin, respectively. (B) Effect of 10^−4^ M cisplatin exposure on intracellular ROS in these cells. (C) Effect of 5 μM menadione on intracellular ROS in these cells. ^*^P<0.05 vs. control cells cultured without cisplatin or menadione. Bars indicate standard deviation. AKR1C2, aldo-keto reductase family 1 member C2; CisR, cisplatin-resistant; ROS, reactive oxygen species.

To elucidate the role of AKR1C2 in cisplatin resistance, the levels of intracellular ROS were determined using an H_2_DCFDA probe under various conditions. Exposure to cisplatin for 2 h increased the level of intracellular ROS in HT1376 cells in a dose-dependent manner (Fig. 3). Significant differences were detected between the ROS levels of HT1376 cells treated without cisplatin and with >10^−6^ M cisplatin. Fig. 4A shows a comparison of relative basal levels of intracellular ROS in HT1376, HT1376-CisR and HT1376-CisR-AKR1C2(−) cells. Intracellular ROS in HT1376-CisR cells was significantly lower than that found in HT1376 cells. Furthermore, AKR1C2 knockdown significantly rescued intracellular ROS levels, although these did not reach the levels found in HT1376 cells. The effects of 10^−4^ M cisplatin exposure for 2 h in the respective cells are shown in Fig. 4B. Cisplatin exposure did not increase the level of intracellular ROS in HT1376-CisR cells, whereas exposure increased the ROS level by 3-fold in HT1376 cells. Silencing AKR1C2 mRNA restored this ROS increase in HT1376-CisR cells.

The effects of 5 μM menadione as an oxidative stressor in the respective cell lines were also examined (Fig. 4C). The addition of menadione to the media increased the ROS levels in HT1376 and HT1376-CisR-AKR1C2(−) cells, but not in HT1376-CisR cells. These data suggest that AKR1C2 expression impairs reactivity against cisplatin-induced oxidative stress in HT1376 cells, thus resulting in cisplatin resistance.

## Discussion

In the present study, AKR1C2 expression was identified only in the cisplatin-resistant human bladder cancer cells. In addition, silencing or inhibition of AKR1C2 restored cisplatin cytotoxicity in these cells, perhaps due to the increase in cisplatin-induced intracellular ROS.

Although cisplatin is widely used for the treatment of advanced and metastatic bladder cancer, the majority of patients relapse with a cisplatin-resistant disease during chemotherapy. The development of chemoresistance remains a major obstacle in the treatment of bladder and other types of cancer (3). The cause of cisplatin resistance has previously been investigated, and proposed mechanisms include reduced intracellular drug accumulation, increased detoxification of the drug by thiol-containing molecules, increased DNA damage repair activities, escape from reactive oxygen species-mediated cytotoxicity and the involvement of apoptosis mediators (14–16). The general consensus is that chemoresistance is multifactorial (i.e., several mechanisms are simultaneously encountered within the same tumor cell) (17–20).

Cisplatin activity is known to generate ROS. For example, cisplatin-induced hearing loss is caused by ROS generation in the cochlea (21). ROS also function as common mediators of apoptosis induced by anticancer drugs. Bragado *et al* (22) reported that the apoptotic activity of cisplatin requires the onset of the p53-mediated p38α mitogen-activated protein kinase pathway through ROS generation. Furthermore, cisplatin-induced apoptosis of cancer cells has been found to act through ROS-dependent Fas aggregation (23). When cancer cells are exposed to high concentrations of ROS by cisplatin treatment, a defense mechanism against intrinsic ROS is activated in these cells. Previous studies have identified several important defense mechanisms that are triggered by cisplatin treatment. The Kelch-like ECH-associated protein 1 (Keap1)/nuclear factor erythroid 2-related factor 2 (Nrf2) system, is one of the most important cellular mechanisms acting against oxidative stressors and electrophiles (24). Keap1 and Nrf2 are oxidative stress sensors and transcription factors for the antioxidant responsive element (ARE). When cells are exposed to stressors such as ROS, Nrf2 is released from the constraint of Keap1 and activates ARE-dependent gene expression (25). The Nrf2/ARE signaling pathway regulates the expression of cytoprotective proteins, including AKR1C2 (25). Although the cytoprotective system is designed to prevent normal cells from becoming cancerous, in a cisplatin-induced ROS-rich environment, cancer cells may hijack the Keap1/Nrf2 system and induce AKR1C2 protein expression as an antioxidant substance. The upregulation of antioxidant capacity in adaptation to intrinsic oxidative stress in cancer cells can result in drug resistance (26).

Previous studies have reported an interaction between AKRs and drug resistance in certain cancer cells. Chen *et al* (6) demonstrated that overexpression of dihydrodiol dehydrogenases (DDHs), which belong to the AKR family, leads to resistance to platinum-based drugs in several human cancer cell lines. These DDH levels are directly responsible for the reduced production of ROS. Chen *et al* (8) also suggested that cisplatin sensitivity appeared to be associated with DDH levels in epithelial lung cancer cell lines. The present study demonstrated that induction of AKR1C2 can be found in cisplatin-resistant human bladder cancer cells and contributes to cisplatin drug resistance. Furthermore, inhibition of AKR1C2 was found to lead to restoration of cisplatin drug sensitivity.

In this study, a cisplatin-resistant human bladder cell line was established from HT1376 cells. Although the biological characteristics of this cell line may not be universal, AKR1C2 expression has frequently been detected in pathological specimens of UC (11). Further studies are required to validate the practical significance of AKR1C2 in bladder cancer. However, we hypothesize that AKR1C2 is one of the biomarkers that indicates cisplatin resistance. In addition, AKR1C2 may be one of the effective molecular targets for rescuing cisplatin sensitivity.
